# Radiation Exposure Predicts Reported Vaccine Adverse Effects in Veterans with Gulf War Illness

**DOI:** 10.3390/ijerph17197136

**Published:** 2020-09-29

**Authors:** Beatrice A. Golomb, Emily Nguyen, Eero Dinkeloo

**Affiliations:** Department of Medicine, School of Medicine, University of California San Diego, La Jolla, CA 92093-0995, USA; emn002@health.ucsd.edu (E.N.); edinkeloo@health.ucsd.edu (E.D.)

**Keywords:** Gulf War illness, adverse effect, vaccine, radiation, pesticide

## Abstract

Most people have no problems when administered vaccines; however, as with all drugs, reported adverse effects (rAEs) do occur. There is a need to better understand the potential predictors of reported vaccine AEs (rVaxAEs), including modifiable (environmental) predictors. Gulf War Veterans (GWV) who have Gulf War illness (GWI) report increased experiences of drug and chemical rAEs, extending to rVaxAEs. GWV provide an opportunity to examine the relationship between their reported exposures and rAEs. Forty one GWV with GWI and 40 healthy controls reported exposure and rAEs to exposure, including for 14 vaccines. Individual and summed vaccine exposures, rVaxAEs, and reported Vaccine AE Propensity (summed rVaxAEs/summed vaccines exposures) were compared in cases vs. controls. Exposure–outcome assessments focused on GWV, using a multivariable regression with robust standard error. More designated vaccines were reported in cases than in controls: 9.0 (2.3) vs. 3.8 (2.3), *p* < 0.0001. The fraction of vaccines received that led to rAEs was ten-fold higher in cases: 0.24 (0.21), vs. 0.023 (0.081), *p* < 0.0001. Multivariable assessment confirmed that radiation and pesticides remained significant statistical predictors of reported Vaccine AE Propensity. Exposure tied to excess rVaxAEs in GWV may contribute to, or underlie, the reported link between rVaxAEs in GWV and later ill health.

## 1. Introduction

Veterans of the 1990–1 Persian Gulf War experienced many exposures; a number reported increased vulnerability to reported adverse effects (rAEs) from a range of drugs and chemicals. Among their exposures were vaccines. While most people receive vaccines without any problems, some experience AEs, and there is cause to understand settings in which this risk may be elevated. rVaxAEs occurred in veterans of the Persian Gulf War and have been tied to later ill health [1].

The high prevalence of many exposures, the often large number of vaccines received by Gulf War Veterans, and their rate of reported vaccine AEs, make them a favorable group in whom to examine the relation of exposures to rVaxAEs [1,2,3,4,5].

This study examines the rate of rVaxAEs reported by veterans with Gulf War illness (GWI), relative to healthy controls. It also examines potential environmental predictors of rVaxAEs in veterans with GWI.

## 2. Methods

### 2.1. Design

The present study assesses the cross-sectional relationships between individual exposures and the propensity to experience AEs to vaccines, via a secondary analysis of a case-control study.

### 2.2. Participants

The 81 participants included 41 veterans with GWI and 40 controls, matched to 40 of the cases on sex, age and ethnicity. An additional case completed the study; recruitment had continued until there were 40 matched pairs, and for this individual GWI case, a matched control had not at that time been identified. Since analyses here are cross-sectional (the primary purpose was not to compare cases to controls, but to look at relationships across and within these groups), the additional case provides additional relevant information on the relationship between exposures and outcomes, and is therefore included.

### 2.3. Cases

To qualify as a GWI case, veterans must have been deployed to the Persian Gulf theater of operations between the dates of 1 August 1990 and 31 July 1991 (inclusive). Veterans were additionally required to both meet at the Centers for Disease Control and Prevention (CDC) and have validated Kansas symptom inclusion criteria for GWI [6,7] as recommended by the Department of Defense (DoD) [8] and the Institute of Medicine/National Academy of Medicine (IOM/NAM) [9]. CDC criteria requires the presence of symptoms for at least 6 months, arising during or after Gulf War participation, in at least two of the three domains of fatigue, mood-cognitive, and musculoskeletal [6]. The more discriminating, more specific Kansas criteria required the symptoms to have been present for at least 6 months, arising during or after Gulf deployment, in at least three of a set of six categories comprising fatigue/sleep, pain, neurological/cognitive/mood, respiratory, gastrointestinal, and dermatologic [7]. For a symptom domain to qualify toward Kansas GWI criteria, the component symptoms must be at least moderate in severity (not mild) and/or there must have been multiple symptoms within the category [7].

### 2.4. Controls

Control participants were nonveterans, meeting neither Kansas nor CDC symptom inclusion criteria for GWI, and additionally not meeting Kansas exclusion criteria (that is, they could not have other health conditions such as lupus or multiple sclerosis (MS) that could have produced symptoms that could have been confused with those of GWI). Controls were selected to match 1:1 with enrolled cases on sex, ethnicity, and age. A half-match for ethnicity was deemed to be qualifying, in recognition of the prevalence of mixed ethnicities. Age matching for matched pairs was within 4 years.

The study in which data were collected was IRB-approved and all participants gave written informed consent.

## 3. Measurements

### 3.1. Demographic Characteristics

Age, sex, ethnicity, and marital status were assessed.

### 3.2. GWI-Relevant Symptoms

Symptoms were gauged by Kansas criteria symptom scores for each Kansas symptom domain.

### 3.3. Exposures

Exposures were elicited via inquiries for a list of general exposures (non-Gulf specific); and for veterans, a list of Gulf-specific exposures. Whether the exposure had been experienced was rated no, unsure, or yes; these were coded as 0, 0.5, and 1, respectively. See supplement for full list of exposures.

### 3.4. Vaccine Exposures

Vaccines about which an inquiry was made included: anthrax (the first military usage was in the Gulf War), botulinum toxoid (usage unique to the Gulf War), cholera, hepatitis A, hepatitis B, twinrx (combined hepatitis A and B), MMR (measles mumps rubella), meningococcal, pertussis, plague, polio, tetanus, typhoid, and yellow fever. The general exposure survey and Gulf-specific exposure survey included many vaccines in common. The vaccine was designated to have been present if it was so designated in either survey. Vaccines assessed only in the general survey were: MMR, hepatitis A and hepatitis B.

A summed vaccine exposure score (vaxEXPtot) summed the exposure ratings across vaccines.

### 3.5. Adverse Effects

Those who rated an exposure as “yes” were asked if they had experienced an AE to the exposure. Response options were again no, unsure, or yes, coded as 0, 0.5, and 1, respectively. A summed vaccine AE score (vaxAEtot) summed responses on the vaccine AE queries.

### 3.6. No Duplicate Counting

Many vaccine queries were included in both the general and Gulf-specific exposure surveys. It is possible that a vaccine could have been received twice, and rAEs could have occurred for each (this may particularly be the case for the anthrax vaccine, which was intended to be a 3-vaccine series per the DoD, and a 6-vaccine series at the time based on its FDA approval). A given vaccine was not counted more than once in vaxEXPtot, nor was an rAE to a given vaccine counted more than once in vaxAEtot. A vaccine, or rVaxAE, was declared to be present if it was so designated on either the general or Gulf survey.

### 3.7. Reported Vaccine AE Propensity

To gauge a proxy for reported Vaccine AE Propensity, a ratio was calculated, of vaxAEtot divided by vaxEXPtot. This approximates the fraction of vaccines received for which rVaxAEs were reported to have been experienced.

### 3.8. Chemical Sensitivity

The study assessed self-rated chemical sensitivity. A question in the Kansas questionnaire rated chemical sensitivity 0–3. Our UCSD binary measure rated self-designated chemical sensitivity as 0 if no chemical sensitivity, 1 if any.

## 4. Analyses

Descriptive statistics characterized case and control demographics, Kansas symptom ratings, vaxEXPtot, reported Vaccine AE Propensity, and the chemical sensitivity measures. T-tests or non-parametric rank-sum tests and chi-squared tests compared characteristics of cases to those of controls for continuous and categorical variables, respectively.

Clinical relations of reported Vaccine AE Propensity were provisionally assessed, evaluating its relation to chemical sensitivity indices and Kansas GWI criteria symptom domains, via correlation. Analyses examining exposure and clinical relations to reported Vaccine AE Propensity focused on Veterans with GWI; these were not meaningful in controls due to few rVaxAEs.

We assessed the provisional relation of individual exposures to reported Vaccine AE Propensity in cases. Shared relationships among exposures within an exposure class were used to guide the generation of candidate composite variables for inclusion in multivariable models (to limit the number of predictors included).

A “main” multivariable model was identified, and reassessed with adjustments for age, sex, ethnicity and other candidate variables. Age-stratified analysis was conducted.

The main model was reassessed, substituting the composite radiation predictor variable with each of its individual radiation exposure (four models, one for each variable).

Analyses used Stata 9.0 and Stata 12.0 (College Station, TX, USA). Two-sided *p* < 0.05 designated statistical significance. Adjustment for multiple comparisons was not undertaken: such adjustment rests on the assumption that chance is the first order explanation for findings [10]. However, stratification was used to examine internal replication.

## 5. Results

Table 1a shows participant characteristics. Mean age was 50 years. About half of the participants were Caucasian, with African Americans the next largest group.

Table 1b shows symptom scores, considering Kansas GWI criteria symptom domains. These are shown for the total sample, and for cases and controls separately. This serves to underscore the high symptom burden experienced by veterans with GWI. Cases are defined by the presence of symptoms in these categories, as described above; while controls are selected for a dearth of such symptoms. Different numbers of symptom queries are asked for the different Kansas criteria domains, providing differences in maximum possible scores, and accounting for material differences in mean scores across domains, in all cases and in controls. The mean summed symptom score for affected veterans is almost 40 and is approximately 75 times higher than the mean score for controls.

Table 2 shows reported vaccines exposures, rVaxAEs, and AEs/exposure in cases and controls, for the 14 queried vaccines; and the summed AEs, exposures, and AEs/exposure variables. Nine of the fourteen vaccines were significantly more frequently reported in veterans with GWI than in controls. Fractions reporting each of the remaining vaccines were also higher in cases, though the difference did not reach significance. Fractions of the individual vaccines received that were reported to have led to AEs were consistently higher in veterans with GWI than in controls (for all Gulf-relevant vaccines), and the summed experience, the fraction of vaccines leading to AEs, was significantly higher among GWI cases. No AEs were reported for 9 of the 14 vaccines in controls, and almost none for the remaining vaccines—precluding meaningful analysis of rVaxAE predictors in controls.

The vaccine associated with the highest proportion experiencing rVaxAEs was the anthrax vaccine, which was consistent with evidence indicating that it is among the most reactogenic of all vaccines [11,12]. The 46% rate of reported AEs may seem high, but it is consistent with findings in studies with an active prospective follow-up, in which rAE rates on the order of 41–92% are reported [13,14,15,16,17] (in part depending on what is counted); as well as the fact that rVaxAEs in Gulf War veterans were tied to later ill health [1], and only veterans with ill health (GWI) were eligible to participate. Participant reporting of the nature of the AE was optional. However, where reports were provided where they did suggest that AE attributions to vaccines were overwhelmingly appropriate. Most were reports of local injection site issues, including lumps on the arm, knots on the arm, burning arm, scab arm, sore arm, large red hot area at the injection site for several days, large welt on skin. For anthrax, one participant cited several problems occurring following injection, including muscle control issues, GI symptoms, and severe headache.

As Table 3 shows, Gulf War veterans—who as part of their deployment received the highest (potential) number of vaccines for any conflict to that date—had more than twice as many of the designated vaccines, on average, than controls. When cases and controls were considered separately, the number of vaccines received was similar by age group (slightly, but not significantly greater in the older group). However, while the number of vaccines received were only slightly greater for older vs. younger, rAEs to vaccines were about twice as prevalent in the older group. (In controls, the fold-difference in rAEs with age is at least as great, though this is based on very small numbers).

Table 4 (right side) shows the relations in cases of reported Vaccine AE Propensity to chemical sensitivity, which was assessed by both a binary and a 4-option rating. The UCSD binary chemical sensitivity rating evaluated symptoms as either absent (0) or present (1). The Kansas question, rating chemical sensitivity on a scale of 0–3 (absent, mild, moderate, and severe), is based on the veteran reporting persistent symptoms over the previous six months. Reported Vaccine AE Propensity did not significantly relate to self-rated chemical sensitivity in this sized sample. The left side of Table 4 shows the mean and standard deviation of the characteristics against which the reported Vaccine AE Propensity was compared in cases vs. controls. Values are seen to be markedly higher in veterans with GWI.

The provisional relations of reported Vaccine AE Propensity to individual exposure were examined (Supplementary Materials Table S1). The relation in all participants is influenced by the fact that case status is linked to exposures, and also to reported Vaccine AE Propensity; the relationships within cases are more relevant. If the exposure relationship with reported Vaccine AE Propensity exhibited a *p*-value ≤ 0.2, the variable was retested for addition to the (later) main model.

Table 5 shows a four-variable model, and the impact of adding age, sex, and chemical sensitivity. Total radiation (adverse), pesticides in living quarters (adverse), and MMR vaccine (favorable) were the leading predictors. Age and sex were not significant predictors after considering exposures (the term “predictor” is used throughout to mean “independent variable” and is not meant to imply causality).

The typhoid vaccine was an apparent predictor. It was not included in the above models in case it was predicted primarily by itself being reactogenic (essentially, predicting the presence of its own AEs). Analogously, MMR vaccine could appear protective, primarily by predicting its own absence of AEs, which might arise since MMR vaccine was generally given before deployment, and prior to deployment exposures that might amplify AE risk.

To assess whether MMR or typhoid vaccines appeared predictive, without predicting themselves, we recreated the reported Vaccine AE Propensity variable excluding both MMR and typhoid vaccine AEs from the numerator, and corresponding exposures from the denominator. We then assessed whether these vaccines predicted the reported Vaccine AE Propensity, to other vaccines (Table 6), which they did. However, typhoid was not predicted in older age, in age stratified analysis. Radiation, pesticides, and MMR remained the more consistent predictors.

Table 7 shows the results, using these three predictors, but substituting each radiation exposure individually, for the composite radiation exposure. Each was a contributor, and except for “other radiation”, each was significant. Pesticides remained significant (or in one case, near-significant) in each model. The MMR vaccine variable coefficient remained stable.

## 6. Discussion

### 6.1. Recap of Findings

In veterans with GWI, radiation exposure, as well as pesticides, appeared to predict greater reported Vaccine AE Propensity.

A possible protective effect of the (presumably earlier) MMR vaccine was not purely related to a lower AE propensity for vaccines given before Gulf theater [18] and associated exposures, i.e., not purely because low rAEs for this vaccine itself disproportionately counted against rVaxAEs. A possible amplifying effect on reported Vaccine AE Propensity by typhoid vaccine was not purely because it was itself a reactogenic vaccine so disproportionately counted toward rVaxAEs. These vaccines’ predictive relationships remained when these vaccines were removed from the numerator and denominator of the reported Vaccine AE Propensity score (that is, when their own rAEs were not among what they were predicting).

### 6.2. Fit with Literature

Reactogenic vaccines are reported to produce oxidative stress [19]. Radiation [20,21,22,23,24,25,26,27,28,29,30,31,32,33,34,35,36,37,38,39,40,41], as well as pesticides [42,43,44,45,46,47,48,49,50,51] (and solvents [52,53]), can depress antioxidant defenses, and by promoting mitochondrial impairment can increase free radical production. This shift in antioxidant defenses versus prooxidant forces might suffice to render some normally innocuous vaccines sufficiently prooxidant to produce symptoms.

It has been reported that multiple vaccinations during deployment to the Gulf [2,3,54,55,56], as well as rVaxAEs among Gulf War personnel [1,18], were linked to future ill health, particularly the chronic multi-symptom health condition that has come to be known as GWI [18]. For the anthrax vaccine, it has been reported that more severe rAEs to the anthrax vaccine are linked to a more severe subsequent health decline [1]. Vaccines received during deployment have been reported to be selectively linked to long term health decline [18], including for the anthrax vaccine [1]. One possibility is that the factors that promote the risk of rVaxAEs—factors that were elevated in Gulf War personnel—also promote the risk of ill health. Radiation and pesticides, in addition to affecting oxidant-antioxidant balance, adversely affect membranes and cause mitochondrial/bioenergetic damage (a mechanism that has been implicated in GWI [57,58]).

Since oxidative stress can promote autoimmunity, it is also possible that vaccines, particularly in settings where they produce AEs via a prooxidant effect (e.g., via adjuvant effects), might promote chronic symptoms through autoimmune mechanisms [59,60,61,62,63]: autoimmune markers are also elevated in GWI [64,65,66,67,68,69,70,71,72,73,74,75,76,77,78,79]. Autoimmune effects have been reported with the reactogenic anthrax vaccine [80,81]. Additionally, in one analysis, female veterans (not confined to the Gulf) who received the anthrax vaccine had elevated rates of MS (RR = 2.14, 95%CI: 1.14, 4.01) [82]. Although the risk ratio for MS in men was 1.0, this is on a backdrop of the “healthy warrior effect”: persons with health problems are disproportionately exempted from deployment to high threat areas, and from vaccination associated with such a deployment, which can produce the appearance of dramatically better outcomes spanning many domains in vaccinated individuals [83] (e.g., hospitalization for cancer is drastically lower in the month or so after anthrax vaccination [83], which is consistent with the expectation that those with advanced cancer, near to the point of requiring hospitalization, were less likely to be deployed to high threat areas, and receive vaccines associated with such a deployment). MS was the sole reported health outcome that did not appear to be *lower* among male military recipients of the anthrax vaccine [82].

Of note, depleted uranium (DU)—a radioactive chemical to which veterans were exposed in the Gulf through DU girded tanks and munitions—has been reported to depress the active form of vitamin D [84], and also vitamin D receptor expression [84]. This is potentially relevant since vitamin D and its receptors are widely reported to play important roles in defense against autoimmune conditions [85,86,87,88,89,90]. (However, part of this relationship could arise from oxidative stressors concurrently depressing levels of active vitamin D, and potentially its receptor, and promoting autoimmunity; that is, the relationship could arise in part from a common cause, rather than from low vitamin D itself promoting autoimmune conditions).

Regarding the apparent protective effect of the MMR vaccine, and the apparent promoting effect of typhoid vaccine, one hypothesis is that both are prooxidant but with differing timing. The former may provide ongoing low-level prooxidation, in a fashion that persistently upregulates endogenous antioxidant systems, a phenomenon termed “oxidative preconditioning.” This can, for instance, specifically protect against exposures that appear relevant to reported Vaccine AE Propensity, such as radiation injury [91,92,93]. Since this was generally given before Gulf participation (and only elicited in the non-Gulf exposure survey), this may have had net protective effects in many by the time of vaccines given in the Gulf.

Radiation exposure has generally not been evaluated as a GWI risk factor. If radiation contributes to rVaxAEs, and if rVaxAEs (which are tied to development of GWI [1,3,18]) causally contribute to GWI, then radiation may be a contributor to GWI.

### 6.3. Limitations

The sample is modest, and findings should be viewed as clearly provisional; however, internal replication for key exposures relations, particularly for radiation and pesticides, enhances confidence in these as contributors. Exposures are remote and recall may be imperfect. Allowing participants to designate an exposure as “unsure” reduces the expected impact of that concern. The botulinum toxoid vaccine was included in both surveys, but will only have been given to Gulf War personnel. It speaks favorably for vaccine reporting that no controls and a minority of cases reported receipt of this vaccine. An important benefit is that exposure characterizations are unlikely to have been altered based on rVaxAE designations, as it was not known that rVaxAE relationships would be examined. Patient reports of drug AEs have been found to show relatively good validity, including assessed against physician reporting [94], and against interviewer administered, elicited AEs [95]. Both on a group and individual level, they have shown dose response and other features that support valid attributions. For example, self-reported AEs from 162 patients closely matched interviewer-determined AEs in a control sample of 1106 patients [95]. Recall bias can arise with self-report, and some might speculate that ill veterans would overreport vaccine AEs. However, reported AEs among GWV were greatest for the anthrax vaccine, at 46%. Prospective follow up of anthrax vaccine recipients provides rAE rates of ~41 to 92%, suggesting against overreporting of AEs by these Veterans [14,15,16,17]. A consideration here is that vaccines in some cases were received contemporaneously: this could complicate the ascertainment of which vaccine was responsible, if an AE was experienced (and indeed, the responsibility could lie with an interaction effect). Whether exposure relationships might extend beyond GWI cases is unknown, as there were too few rVaxAEs among the control group to allow a meaningful assessment in that group; however, in an analysis of Total AE Propensity (not shown, available on request), which shared common risk factors, exposure-relationships were reproduced in controls, both supporting the validity of veterans’ reports, and suggesting that similar relationships may obtain in controls. Though exposure assessments were extensive, they were not comprehensive and it is possible that important exposure(s) were missed. In the case of radiation exposure, participants will have been aware, in general, of x-rays, other medical radiation, work with radioactive chemicals, and exposure in-theater to depleted uranium shrapnel or aerosolization (e.g., friendly fire episodes). However, there could be radiation exposures of which participants were unaware.

### 6.4. Implications

Findings should be reexamined in a larger sample of Gulf War Veterans—and perhaps in prospective studies of high-exposure groups, and/or retrospective studies of persons with rVaxAEs. More must be learned about the time sensitivity of exposure–outcome relationships—that is, the extent to which AE-influencing exposures, if verified, must be close in time to the vaccine exposures to be relevant to prospects for rVaxAEs (and whether such relationships cohere with, for instance, prooxidant—antioxidant status). Information was not collected to enable this assessment. Until such factors are better understood, consideration might be given to planning vaccine delivery in military personnel well before significant exposures to factors like radiation or pesticides, when these can be predicted, or at least during intervals in which exposure levels are low.

## 7. Conclusions

This analysis takes a small first step in assessing what factors may have led some veterans of the Persian Gulf War to have had unexpectedly high rates of vaccine-adverse reactions. It takes a small first step towards identifying environmental (and thus potentially modifiable) risk factors for rVaxAEs, which is knowledge that may assist in optimizing the timing and safety of vaccine administration.

## Figures and Tables

**Table 1:** Demographics and Kansas Criteria Symptom Scores * of Study Participants (n = 81).

**1a. Demographic Features**	**All** **n = 81**	**Cases** **n = 41**	**Controls** **n = 40**	***p* for Difference** **(Case vs. Control)**
Age (in Years), Mean (SD)	49.8 (7.5)	50.1 (7.6)	49.5 (7.5)	0.68
Male, %	92.6	92.7	92.5	0.98
Ethnicity, %				
Caucasian	54.3	53.7	55.0	0.90
African American	21.0	22.0	20.0	0.83
Latino	14.8	14.6	15.0	0.96
Asian	7.4	7.3	7.5	0.98
Native American	2.4	2.4	2.5	0.99
Married, %	51.8	65.9	38.1	0.011
**1b. Kansas Criteria Domain** **Symptom Scores**	**All** **n = 81**	**Cases** **n = 41**	**Controls** **n = 40**	***p* for Difference** **(Case vs. Control)**
Total Fatigue	3.88 (4.33)	7.49 (3.20)	0.175 (0.501)	<0.0001
Total Pain	2.73 (3.14)	5.27 (2.48)	0.125 (0.404)	<0.0001
Total Neurological	9.47 (11.0)	18.5 (8.31)	0.175 (0.501)	<0.0001
Total Skin	0.95 (1.67)	1.85 (1.96)	0.025 (0.158)	<0.0001
Total Gastrointestinal	1.86 (2.44)	3.66 (2.29)	0.025 (0.158)	<0.0001
Total Respiratory	1.19 (1.85)	2.32 (2.04)	0.025 (0.158)	<0.0001
Summed Kansas Domain Scores	19.6 (22.5)	39.1 (16.1)	0.524 (0.943)	<0.0001

SD = Standard Deviation; n = number; *p* = probability value. * Kansas Criteria Symptom Scores rate severity of symptoms over the last six months in each of six different domains. Thirty symptoms are each rated from 0–3 (absent, mild, moderate, severe).

**Table 2:** Reported Vaccine Exposure and Reported Vaccine Adverse Effects (rAEs) are More Common in Cases than Controls (n = 81).

Vaccine Type			Vaccine Exposures			Reported Vaccine AEs			Reported Vaccine AE per Exposure		
Vaccine	Incl Gen	Incl Gulf	Case (n = 41) Mean (SD)	Control (n = 40) Mean (SD)	*p* Rank- Sum	Case (n = 41) Mean (SD)	Control (n = 40) Mean (SD)	*p* Rank- Sum	Case AE/Exp (n = 41) Mean (SD)	Control (n = 40) Mean (SD)	*p* Rank- Sum
Anthrax	*√*	*√*	0.84 (0.32)	0.088 (0.27)	<0.0001	0.41 (0.31)	0	<0.0001	0.46 (0.30)	0	0.0037
Botulinum toxoid	*√*	*√*	0.43 (0.33)	0	<0.0001	0.073 (0.18)	0	0.013	0.14 (0.30)	N/A	N/A
Cholera	*√*	*√*	0.55 (0.37)	0.05 (0.22)	<0.0001	0.15 (0.23)	0	0.0002	0.23 (0.34)	0	0.30
Hepatitis A	*√*		0.46 (0.44)	0.33 (0.43)	0.14	0.16 (0.24)	0.05 (0.16)	0.22	0.20 (0.32)	0.05 (0.16)	0.18
Hepatitis B	*√*		0.59 (0.45)	0.48 (0.47)	0.28	0.11 (0.21)	0.031 (0.13)	0.17	0.14 (0.28)	0.031 (0.13)	0.15
TwinRx (hepA + hepB)	*√*	*√*	0.57 (0.36)	0.13 (0.27)	<0.0001	0.12 (0.22)	0	0.0009	0.20 (0.33)	0	0.08
MMR	*√*		0.81 (0.37)	0.69 (0.42)	0.12	0.14 (0.23)	0.04 (0.2)	0.025	0.15 (0.23)	0.04 (0.20)	0.022
Meningococcal	*√*	*√*	0.57 (0.35)	0.088 (0.19)	<0.0001	0.11 (0.21)	0	0.0018	0.16 (0.29)	0	0.13
Pertussis	*√*	*√*	0.61 (0.36)	0.33 (0.46)	0.0024	0.12 (0.22)	0.013 (0.079)	0.0043	0.16 (0.27)	0.036 (0.13)	0.10
Polio	*√*	*√*	0.72 (0.35)	0.53 (0.48)	0.077	0.15 (0.26)	0.025 (0.16)	0.0029	0.18 (0.30)	0.043 (0.21)	0.021
Plague	*√*	*√*	0.55 (0.35)	0.038 (0.17)	<0.0001	0.12 (0.22)	0	0.0009	0.20 (0.33)	0	0.37
Tetanus	*√*	*√*	0.93 (0.21)	0.80 (0.39)	0.16	0.28 (0.25)	0	<0.0001	0.33 (0.31)	0	<0.0001
Typhoid	*√*	*√*	0.71 (0.33)	0.15 (0.34)	<0.0001	0.21 (0.27)	0	<0.0001	0.27 (0.35)	0	0.034
Yellow Fever	*√*	*√*	0.72 (0.35)	0.088 (0.25)	<0.0001	0.18 (0.24)	0	<0.0001	0.21 (0.25)	0	0.073
**Total**	*√*	*√*	9.0 (3.2)	3.8 (2.3)	<0.0001	2.2 (2.1)	0.088 (0.32)	<0.0001	0.24 (0.21)	0.023 (0.081)	<0.0001

rAE = reported adverse effect; Incl Gen = included in general survey; Incl Gulf = included in Gulf-specific survey; SD = Standard Deviation. Exp = Exposure; MMR = Measles, Mumps, and Rubella Vaccine.

**Table 3:** Vaccine Summary Variables Among Cases and Controls, Stratified by Age (n = 81).

Vaccine Summary Variables	CASES (n = 41)				CONTROLS (n = 40)			
	Age < 48 (n = 20)	Age > 48 (n = 21)	*p* for Difference (Ranksum)	Fold-Difference	Age < 48 (n = 21)	Age > 48 (n = 19)	*p* for Difference (Ranksum)	Fold-Difference
	Mean (SD)	Mean (SD)			Mean (SD)	Mean (SD)		
Age (in years)	44 (1.6)	56 (5.4)	<0.0001	n/a	43 (2.7)	56 (5.2)	<0.0001	n/a
Summed Vaccine Exposure Score (vaxEXPtot)	8.7 (3.2)	9.4 (3.2)	0.43	1.1	3.5 (2.2)	4.0 (2.4)	0.71	1.1
Summed Vaccine AE Score (vaxAEtot)	1.5 (1.9)	2.8 (2.2)	0.015	1.9	0.048 (0.22)	0.13 (0.40)	0.48	**2.7**
Vaccine AE Propensity *	0.17 (0.17)	0.31 (0.22)	0.029	1.8	0.015 (0.066)	0.032 (0.095)	0.52	**2.1**

AE = Adverse Effect; SD = Standard Deviation * Vaccine Adverse Effect Propensity = ratio of summed vaccine adverse effects to summed exposures (vaxAEtot/vaxEXPtot).

**Table 4:** Comparison of Chemical Sensitivity Scores between Cases and Controls (n = 81); and their Relation to Reported Vaccine AE Propensity Among Cases (n = 41).

Chemical Sensitivity Rating	Case (n = 41)	Control (n = 40)	*p* for Difference (Case vs. Control)	Reported Vaccine AE Propensity Relation ^††^ among Cases (n = 41)		
				Chemical Sensitivity Rating	r	*p*
UCSD Binary CS, proportion with CS	0.54 (0.50)	0.025 (0.16)	<0.0001 *	UCSD Binary CS	0.29	0.068
Kansas CS (rated 0–3), Mean (SD)	1.1 (1.1)	0.025 (0.16)	<0.0001 ^†^	Kansas CS (rated 0–3)	−0.0017	0.99

AE = Adverse Effect; SD = Standard Deviation; *p* = probability value; r = correlation coefficient. UCSD Binary CS = University of California San Diego binary rating system for chemical sensitivity (0 if absent, 1 if present); Kansas CS = Kansas questionnaire chemical sensitivity rating (rated 0–3, based on absent, mild, moderate, or severe symptoms).* Chi-square test; ^†^ Sign rank test; ^††^ Spearman correlation.

**Table 5:** Multivariate Models Predicting Reported Vaccine Adverse Effect (AE) Propensity (n = 40).

Main Model R^2^ = 0.46, *p* < 0.0001			Age–Sex Adjusted Model R^2^ = 0.50, *p* = 0.0001			Main Model Adjusted for Chemical Sensitivity R^2^ = 0.46, *p* < 0.0001		
Variable	β (SE)	*p*	Variable	β (SE)	*p*	Variable	β (SE)	*p*
Total Radiation	0.035 (0.0098)	0.001	Total Radiation	0.037 (0.0097)	0.001	Total Radiation	0.034 (0.011)	0.003
Pesticides Sprayed in Living Quarters	0.14 (0.060)	0.021	Pesticides Sprayed in Living Quarters	0.14 (0.058)	0.021	Pesticides Sprayed in Living Quarters	0.14 (0.061)	0.023
MMR Vaccine	−0.22 (0.097)	0.034	MMR Vaccine	−0.21 (0.090)	0.028	MMR Vaccine	−0.21 (0.10)	0.038
Solvents	0.087 (0.050)	0.087	Solvents	0.084 (0.046)	0.076	Solvents	0.086 (0.053)	0.12
			Male	−0.041 (0.027)	0.14	Chem Sensitivity ^a^	0.0056 (0.059)	0.93
			Age (in Years)	0.0052 (0.0036)	0.15			

Multivariable regressions with robust standard errors. β = beta (regression coefficient); SE = standard error; AE = adverse effect; MMR = Measles, Mumps, and Rubella; ^a^ Chemical Sensitivity was assessed using the UCSD Binary Rating (0 for absent, 1 for present).

**Table 6:** MMR and Typhoid Vaccines as Independent Predictors ^a^ of Reported Vaccine Adverse Effect Propensity Score (n = 40).

Main Model n = 40 R^2^ = 0.52, *p* < 0.0001			Age-Stratified Model					
			<52 Years of Age n = 25 R^2^ = 0.54, *p* = 0.0022			≥52 Years of Age n = 15 R^2^ = 0.55, *p* = 0.023		
Variable	β (SE)	*p*	Variable	β (SE)	*p*	Variable	β (SE)	*p*
Total Radiation	0.038 (0.0092)	<0.001	Total Radiation	0.039 (0.011)	0.002	Total Radiation	0.051 (0.018)	0.017
Pesticides Sprayed in Living Quarters	0.15 (0.051)	0.005	Pesticides Sprayed in Living Quarters	0.14 (0.077)	0.089	Pesticides Sprayed in Living Quarters	0.16 (0.078)	0.069
MMR Vaccine	−0.29 (0.095)	0.004	MMR Vaccine	−0.23 (0.12)	0.066	MMR Vaccine	−0.35 (0.15)	0.045
Typhoid Vaccine	0.18 (0.057)	0.003	Typhoid Vaccine	0.17 (0.083)	0.051	Typhoid Vaccine	0.10 (0.10)	0.34

Multivariable regression with robust standard error. β = regression coefficient; SE = standard error; AE = adverse effect; MMR = Measles, Mumps, and Rubella; ^a^ The models in this table have excluded MMR and Typhoid Vaccines from the numerator and denominator in calculation of reported Vaccine Adverse Effect Propensity.

**Table 7:** Multivariable Models Predicting Reported Vaccine Adverse Effect Propensity—Reassessed by Individual Radiation Exposure (n = 40).

Included Variables	Radioactive Chemicals R^2^ = 0.43, *p* = 0.0002		Radiation Therapy R^2^ = 0.36, *p* = 0.0004		X-ray Radiation R^2^ = 0.36, *p* < 0.0001		Other Radiation R^2^ = 0.30, *p* = 0.0011	
	β (SE)	*p*	β (SE)	*p*	β (SE)	*p*	β (SE)	*p*
Radiation Exposure ^a^	0.23 (0.075)	0.004	0.29 (0.13)	0.032	0.15 (0.048)	0.003	0.099 (0.062)	0.12
Pesticides Sprayed in Living Quarters	0.14 (0.067)	0.043	0.18 (0.067)	0.013	0.21 (0.065)	0.003	0.18 (0.069)	0.014
MMR Vaccine	−0.20 (0.095)	0.047	−0.19 (0.11)	0.077	−0.19 (0.10)	0.079	−0.19 (0.11)	0.082

Multivariable regression with robust standard error. β = beta; variable’s regression coefficient; SE = standard error; MMR = Measles, Mumps, and Rubella. ^a^ Radiation exposure is represented by one individual exposure in each model, rather than the composite score of all four combined.

## Data Availability

Data will be provided on request with the following proviso. Data to allow reproduction of Table 1a,b participant characteristics will be provided in separate databases from a database to allow assessment of the main models in Table 5 and Table 6, to reduce any risk that participants will be identifiable.

## Supplementary Materials

The following are available online at https://www.mdpi.com/1660-4601/17/19/7136/s1, Table S1a: Exposure correlations to Reported Vaccine Adverse Effect (AE) Propensity, asked of all participants (n = 81), Table S1b: Gulf War Specific Exposure Correlations with Reported Vaccine AE Propensity Among Cases (n = 41).
