# Supplementary material for: Radiation Exposure Predicts Reported Vaccine Adverse Effects in Veterans with Gulf War Illness
Source: Int J Environ Res Public Health. 2020 Sep 29;17(19):7136. doi: 10.3390/ijerph17197136 (PMC7579364; doi:10.3390/ijerph17197136)
Supplement: Supplementary file 1 [file ijerph-17-07136-s001.zip › VAE Supplement/REVISED_ijerph-920395-supplementary.docx]

**Supplement**

| **Table S1a. Exposure correlations to Reported Vaccine Adverse Effect (AE) Propensity, asked of all participants (n=81)** | | | | | | |
| --- | --- | --- | --- | --- | --- | --- |
| Abbreviation* | Exposure Name | Correlation to reported Vaccine AE Propensity – All  (n = 81) | | Correlation to reported Vaccine AE Propensity – Cases (n = 41) | | Significance if Added to the Main Analysis**  Cases  (n = 41) |
|  |  | r | p | r | p |  |
| Dryclean | Dry cleaned clothing | 0.24 | 0.025 | -0.16 | 0.35 |  |
| Fabric softener | Fabric softeners, dryer sheets | -0.042 | 0.71 | -0.15 | 0.37 |  |
| Chemical cleaners | Chemical cleaning products | 0.22 | **0.052** | 0.31 | **0.049** | 0.32 |
| Burning fuel | Burning fuels | 0.46 | **<0.0001** | 0.21 | **0.20** | 0.71 |
| Carbon monoxide | Carbon monoxide | 0.36 | **0.0013** | 0.35 | **0.025** | 0.69 |
| Dslfume_ge | Diesel or Petrochemical Fumes | 0.38 | **0.0006** | 0.12 | 0.46 |  |
| Fuelfume_ege |  | 0.47 | **<0.0001** | 0.10 | 0.52 |  |
| Kerosene | Kerosene | 0.43 | **0.0001** | 0.27 | **0.082** | 0.39 |
| Jet fuel | Jet fuel | 0.43 | **0.0001** | 0.21 | **0.18** | 0.98 |
| Fuel Storage | Stored fuels | 0.30 | **0.0069** | 0.18 | 0.25 |  |
| Petro | Petroleum products | 0.28 | **0.012** | 0.12 | 0.46 |  |
| Deisel skin  Fuelskin_ege | Diesel or petrochemical fuel on skin | 0.20  0.36 | **0.079**  **0.0011** | -0.079  0.070 | 0.62  0.66 |  |
| Degreasing solution | Degreasing solutions | 0.36 | **0.0014** | 0.37 | **0.016** | 0.84 |
| Solvthin | Other solvents | 0.33 | **0.0036** | 0.35 | **0.024** | 0.46 |
| Acetone | Acetone | 0.35 | **0.0017** | 0.38 | **0.013** | 0.31 |
| Solvents | Solvents | 0.40 | **0.0002** | 0.39 | **0.012** | 0.087 |
| Paint_ge  Paint_ege | Paint, paint strippers | 0.39  0.35 | **0.0005**  **0.0018** | 0.44  0.33 | **0.0036**  **0.037** | 0.18  0.54 |
| Napthas | Napthas | 0.24 | **0.035** | 0.22 | 0.16 | 0.94 |
| Deet_ge | DEET | 0.35 | **0.0019** | 0.060 | 0.71 |  |
| Dursban | Dursban | 0.29 | **0.011** | 0.18 | 0.27 |  |
| Fleatick | Flea or tick medicine | 0.17 | 0.13 | 0.058 | 0.72 |  |
| Lice treatment | Head lice treatment | 0.18 | 0.12 | 0.16 | 0.33 |  |
| Organochlorine | Organochlorines | 0.17 | 0.14 | 0.059 | 0.71 |  |
| Organophosphate | Organophosphates | 0.28 | **0.012** | 0.16 | 0.31 |  |
| Flyspray | Fly spray | 0.28 | **0.012** | 0.22 | **0.17** | 0.61 |
| Pestskin_ge  Pestskin_ege | Pesticide cream or spray on skin | 0.28  0.31 | **0.013**  **0.0051** | -0.0095  -0.15 | **0.95**  **0.34** |  |
| Pestcloth_ge  Pestcloth_ege | Pesticides on clothing or bedding | 0.44  0.50 | **0.0001**  **<0.0001** | 0.22  0.26 | **0.17**  **0.10** | 0.38  0.37 |
| Pyrethroid | Pyrethroid pesticides/pest repellants | 0.37 | **0.0010** | 0.22 | **0.18** | 0.90 |
| Livepest | Saw the area in which you lived fogged or sprayed with pesticides | 0.28 | **0.013** | 0.22 | **0.17** | 0.60 |
| Raid | Raid | 0.28 | **0.014** | 0.30 | **0.057** | 0.22 |
| Black flag | Black Flag | 0.31 | **0.0060** | 0.28 | **0.084** | 0.081 |
| Moth repellent | Moth repellants/moth balls | 0.32 | **0.0038** | 0.33 | **0.036** | 0.68 |
| Insect repellent | Insect repellent | 0.48 | **<0.0001** | 0.36 | **0.021** | 0.10 |
| Carbamate pest | Carbamate pesticides | 0.39 | **0.0005** | 0.29 | **0.073** | 0.87 |
| Other pesticide | Other pesticides/insecticides | 0.54 | **<0.0001** | 0.41 | **0.0070** | 0.17 |
| Animal spray | Animal sprays | 0.41 | 0.0002 | 0.40 | **0.010** | 0.88 |
| Pest treatment at work | Regular pesticide treatment at place of work | 0.26 | **0.020** | 0.25 | **0.12** | 0.67 |
| Wood treatment | Wood treatment | 0.13 | 0.26 | 0.078 | 0.63 |  |
| Roundup | Roundup (herbicide) | 0.26 | **0.020** | 0.16 | 0.31 |  |
| Regular herbicide at work | Regular herbicide treatment at place of work | 0.15 | 0.20 | 0.041 | 0.80 |  |
| Other herbicides | Other herbicides | 0.32 | **0.0040** | 0.21 | **0.19** | 0.57 |
|  |  |  |  |  |  |  |
| Selenium | Selenium | 0.37 | **0.0007** | 0.21 | **0.18** | 0.22 |
| Copper | Copper | 0.21 | **0.061** | 0.042 | 0.79 |  |
| Arsenic | Arsenic | 0.34 | **0.0020** | 0.16 | 0.32 |  |
| Chromium | Chromium | 0.18 | 0.12 | 0.13 | 0.42 |  |
| Cobalt | Cobalt | 0.30 | **0.0069** | 0.19 | 0.24 |  |
| Lead | Lead | 0.28 | **0.013** | -0.0075 | 0.96 |  |
| Manganese | Manganese | 0.38 | **0.0006** | 0.24 | **0.13** | 0.44 |
| Iron | Iron | 0.43 | **0.0001** | 0.32 | **0.041** | 0.36 |
| Mercury | Mercury | 0.30 | **0.0073** | 0.30 | **0.054** | 0.23 |
| Cadmium | Cadmium | 0.37 | **0.0007** | 0.27 | **0.087** | 0.54 |
| Zinc | Zinc | 0.39 | **0.0005** | 0.27 | **0.082** | 0.84 |
| Thallium | Thallium | 0.46 | **<0.0001** | 0.46 | **0.0026** | 0.21 |
|  |  |  |  |  |  |  |
| Radiation therapy | Radiation treatment for cancer or other conditions | 0.30 | **0.0083** | 0.37 | **0.016** | **In main model, as part of composite variable “totrad” or total radiation.** |
| Other radiation | Other radiation | 0.45 | **<0.0001** | 0.29 | **0.068** |  |
| x-ray radiation | X-rays, radiation | 0.31 | **0.0052** | 0.31 | **0.052** |  |
| Radioactive chemicals | Radioactive chemicals | **0.51** | **<0.0001** | 0.49 | **0.0012** |  |
| Totrad | Summed radiation exposures | 0.46 | **<0.0001** | 0.41 | **0.0080** | **0.001** |
|  |  |  |  |  |  |  |
| Aerosol | Aerosol sprays | 0.21 | **0.067** | 0.18 | 0.25 |  |
| Air freshener | Air fresheners | 0.27 | **0.016** | 0.28 | **0.075** | 0.36 |
| Asbestos | Asbestos | 0.41 | 0.0002 | 0.18 | 0.25 |  |
| Auto products | Automotive products | 0.20 | 0.083 | 0.22 | 0.17 |  |
| Personal care products | Personal care | 0.14 | 0.21 | 0.23 | 0.15 |  |
| Cologne | Cologne | 0.20 | 0.085 | 0.33 | **0.034** | 0.25 |
| Diet soda | Drinking diet soda | 0.082 | 0.47 | 0.20 | 0.22 |  |
| Drywall | Drywall dust | 0.18 | 0.12 | 0.15 | 0.36 |  |
| Mold fungus | Mold or fungus | 0.29 | 0.0088 | 0.20 | 0.22 |  |
| Office exposures | Office exposures | 0.23 | 0.046 | 0.31 | **0.051** | 0.47 |
| Makeup | Regular make-up use | -0.029 | 0.80 | -0.16 | 0.31 |  |
| Sunscreen_ge  Sunscreen_ege | Use of suntan lotion or sunscreen | 0.088  0.21 | 0.44  **0.065** | 0.055  0.030 | 0.73  0.85 |  |
| H2otxche_ge  H2otxchem_ege | Water treatment chemicals | 0.18  **0.38** | 0.11  **0.0007** | 0.083  0.20 | 0.61  0.21 |  |
|  |  |  |  |  |  |  |
| Amalaria_ge  Amalaria_ege | Antimalarial drugs | 0.28  0.39 | 0.012  0.0004 | -0.044  0.048 | 0.78  0.77 |  |
| Fluroq_ge  Fluroq_ege | Ciprofloxacin or other fluoroquinolone | 0.32  0.29 | 0.0039  0.0091 | 0.17  0.044 | 0.29  0.78 |  |
| Chemo | Chemotherapy | **0.35** | **0.0016** | **0.30** | **0.053** | 0.012 |
| Doxycycline | Doxycycline | 0.29 | 0.011 | -0.019 | 0.91 |  |
| Botox | Botox vaccine | 0.12 | 0.29 | -0.0048 | 0.98 |  |
| Anthrax_ge  Anthrax_ege | Anthrax vaccine | **0.50**  **0.50** | **<0.0001**  **<0.0001** | 0.22  0.19 | **0.18**  0.25 | 0.20 |
| Btvac_ge  Btvac_ege | Botulinum toxoid or “BT” vaccine | 0.36  **0.41** | 0.0011  **0.0002** | 0.12  0.055 | 0.47  0.73 |  |
| Cholera_ge  Cholera_ege | Cholera vaccine | 0.32  0.35 | 0.0038  0.0017 | 0.11  0.0060 | 0.50  0.97 |  |
| Hepatitis A vaccine | Hepatitis A vaccine | 0.11 | 0.34 | 0.083 | 0.61 |  |
| Hepatitis B vaccine | Hepatitis B vaccine | -0.02 | 0.85 | -0.068 | 0.67 |  |
| Twinrx_ge  Hepab_ege | TwinRx (combined HepA and HepB) vaccine | 0.081  0.22 | 0.49  **0.048** | 0.0032-0.14 | 0.98  0.38 |  |
| Immglob_ge  Immglob_ege | Immune globulin (gamma globulin) vaccine | 0.34  0.46 | 0.0022  <0.0001 | 0.11  0.20 | 0.49  0.21 |  |
| Meningo_ge  Meningo)ege | Meningococcal vaccine | 0.36  0.38 | 0.0011  0.0006 | 0.19  0.053 | 0.24  0.74 |  |
| MMR – measles mumps rubella vaccine | MMR (measles, mumps, rubella) vaccine | -0.067 | 0.56 | -0.29 | **0.067** | **0.034** |
| Pertuss_ge  Pertuss_ege | Pertussis vaccine | 0.18  0.18 | 0.11  0.12 | 0.13  -0.0080 | 0.42  0.96 |  |
| Plague vaccine  Plague_ege | Plague vaccine | 0.29 | 0.011 | 0.017  -0.15 | 0.92  0.34 |  |
| Polio_ge  Polio_ege | Poliomyelitis vaccine | 0.12  0.12 | 0.28  0.29 | 0.036  -0.088 | 0.82  0.58 |  |
| Tetanus_ge  Tetanus_ege | Tetanus vaccine | -0.078  -0.096 | 0.50  0.40 | -0.35  -0.44 | **0.027**  **0.0042** | 0.38  0.21 |
| Typhoid_ge  Typhoid_ege | Typhoid vaccine | 0.39  0.41 | **0.0005**  **0.0002** | 0.22  0.18 | 0.17  0.25 | **0.003** |
| Yfever_ge  Yfever_ege | Yellow fever vaccine | 0.36  0.39 | **0.0011**  **0.0004** | 0.032  -0.0015 | 0.84  0.99 |  |
| *Abbreviations ending in “_ge” were asked on the general exposure survey  *Abbreviations ending in “_ege” are a combination of the general and Gulf War specific survey  **Variables were included for testing in the main model if univariate correlation to reported Vaccine AE Propensity exhibited p <0.20 | | | | | | |

| **Table S1b. Gulf War Specific Exposure Correlations with Reported Vaccine AE Propensity Among Cases (n=41)** | | | | |
| --- | --- | --- | --- | --- |
| Abbreviation | Exposure Name | r | p-value | Significance if Added to the Main Analysis*  Cases (n = 41) |
| NBC suits | NBC suits | 0.068 | 0.67 |  |
| Chemical attack | Chemical or nerve gas attack | -0.13 | 0.43 |  |
| Chemical alarm | Heard chemical alarms sounded | 0.12 | 0.47 |  |
| Gas mask | Use of gas masks | 0.10 | 0.52 |  |
| Spray vehicle | Respraying vehicles | 0.099 | 0.54 |  |
| Chemical agent resistant compound paint | Used or came into contact with freshly applied CARC paint | -0.044 | 0.78 |  |
| Paints_e | Other paints or solvents | 0.096 | 0.55 |  |
| Fuel on skin | Diesel or petrochemical fuel on skin | 0.066 | 0.68 |  |
| Fuel fumes | Diesel or petrochemical fumes | -0.13 | 0.40 |  |
| Exhaust generator | Exhaust from heaters or generators | 0.19 | 0.24 |  |
| Munition fumes | Fumes from munitions | -0.14 | 0.39 |  |
| Inhale smoke | Inhaled smoke from oil-well fires | 0.13 | 0.40 |  |
| Saw smoke (from oil fires) | Saw smoke from oil-well fires | 0.11 | 0.50 |  |
| Burn jet fuel | Jet fuel burned in tent heaters | 0.30 | **0.060** | 0.71 |
| Burning rubbish/ burn pits | Burning rubbish or feces | 0.23 | 0.15 |  |
| Flea collars | Wore a flea collar | -0.0033 | 0.98 |  |
| Insect repellent | Insect repellents | -0.021 | 0.90 |  |
| Saw pesticide sprayed | Saw the area in which you lived fogged or sprayed with pesticides | 0.16 | 0.33 |  |
| Pesticide sprayed in quarters | Sprayed quarters with pesticides | 0.40 | **0.0086** | **0.049** |
| Pesticide treatment of uniform | Wore a uniform treated with pesticides | 0.22 | **0.17** | 0.46 |
| Permethrin treated uniform | Wore permethrin-impregnated uniforms | -0.10 | 0.52 |  |
| Pesticides on skin | Used pesticide cream or spray on skin | -0.053 | 0.74 |  |
| Pesticides on clothing | Pesticides on clothing or bedding | 0.098 | 0.54 |  |
| Pesticide handling | Pesticides handling | 0.23 | **0.14** | 0.67 |
| Personal pesticide | Personal pesticides | 0.20 | **0.21** | 0.71 |
| Mustard gas | Mustard gas | 0.22 | **0.18** | 0.61 |
|  |  |  |  |  |
| Ate local food | Local food | 0.14 | 0.39 |  |
| Got food poisoning | Getting food poisoning | 0.21 | **0.18** | 0.62 |
| Unit got food poisoning | Food poisoning in one’s unit | 0.21 | **0.18** | 0.81 |
| Diet soda | Drinking diet soda | -0.057 | 0.73 |  |
| Drink water from desert bag | Drinking water from a desert bag | -0.11 | 0.48 |  |
| Bad water | Drinking contaminated water | 0.29 | **0.066** | 0.67 |
| Bath in local pond/ water | Bathing in local pond or river or Gulf waters | -0.17 | 0.30 |  |
| Water treatment chemicals | Water treatment chemicals | -0.024 | 0.88 |  |
| Antimalarials | Antimalarial drugs | 0.026 | 0.87 |  |
| Ciprofloxacin | Took ciprofloxacin | -0.028 | 0.86 |  |
| Doxycycline | Took doxycycline | 0.20 | **0.20** | 0.44 |
| Pyridostigmine bromide pills | Took pyridostigmine pills | -0.0024 | 0.99 |  |
| No sleep | Had less than 4 hours of sleep in a 24 hour period | 0.018 | 0.91 |  |
|  |  |  |  |  |
| Tented accommodations | Tented accommodations | -0.15 | 0.36 |  |
| Temporary wooden structure | Lived in temporary wooden structure | 0.12 | 0.47 |  |
| Sandstorm | Sandstorms | 0.10 | 0.52 |  |
| Sunscreen | Use of suntan lotion or sunscreen | -0.029 | 0.86 |  |
|  |  |  |  |  |
| Arm immunization | Received one or more immunizations in the arm while in theater | 0.27 | **0.087** | (related to individual vaccines, so not assessed in main model) |
| Butt immunization | Received one or more immunizations in the buttocks while in theater | 0.35 | **0.024** |  |
| Anthrax vaccine | Anthrax vaccine | 0.19 | 0.25 |  |
| Botulinum toxoid vaccine | Botulism vaccine | 0.060 | 0.71 |  |
| Cholera vaccine | Cholera vaccine | 0.048 | 0.76 |  |
| Hepatitis A + B vaccine | Hepatitis A, B vaccine | -0.11 | 0.49 |  |
| Immune globulin | Immune globulin vaccine | 0.21 | **0.18** | 0.13 |
| Meningococ vaccine | Meningococcal vaccine | 0.12 | 0.45 |  |
| Pertussis vaccine | Pertussis vaccine | 0.089 | 0.58 |  |
| Plague vaccine | Plague vaccine | -0.12 | 0.47 |  |
| Polio vaccine | Poliomyelitis vaccine | 0.11 | 0.48 |  |
| Tetanus vaccine | Tetanus vaccine | -0.057 | 0.72 |  |
| Typhoid vaccine | Typhoid vaccine | 0.22 | **0.16** | 0.23 |
| Yellow fever vaccine | Yellow fever vaccine | 0.10 | 0.52 |  |
|  |  |  |  |  |
| Contact with POWs | Came into contact with prisoners of war | -0.19 | 0.23 |  |
| Combat air | Was directly involved in air combat | 0.10 | 0.54 |  |
| Combat ground | Was directly involved in ground combat | 0.021 | 0.90 |  |
| Combat injury | Combat-related injury | 0.29 | **0.062** | 0.51 |
| Direct contact destroyed enemy vehicle (so, DU) | Came into direct contact with destroyed enemy vehicles | 0.084 | 0.60 |  |
| Saw destroyed enemy vehicle | Saw destroyed enemy vehicles | 0.052 | 0.75 |  |
| Direct contact vehicle exposed to friendly fire | Came into direct contact with American vehicles hit by friendly fire | 0.17 | 0.30 |  |
| Saw dismembered bodies | Saw dismembered bodies | -0.046 | 0.77 |  |
| Saw Americans killed | Saw American or Allied troops who had been badly wounded or killed | 0.11 | 0.48 |  |
| Saw Iraqis killed | Saw Iraqis/civilians badly wounded or killed | -0.064 | 0.69 |  |
| Dir contact dead animals | Came into direct contact with dead animals | 0.18 | 0.26 |  |
| Saw dead animals | Saw dead animals | 0.043 | 0.79 |  |
| Saw death | Witness anyone dying | -0.11 | 0.51 |  |
| Came under small arms fire | Came under small arms fire | -0.22 | 0.16 | 0.25 |
| Danger | Danger/direct combat | -0.26 | 0.10 | **0.004** |
| Scud | Had SCUD missile explode within one mile of you | -0.013 | 0.94 |  |
| Medical attention | Medical attention | 0.13 | 0.43 |  |
| *Variables were included for testing in the main model if univariate correlation to reported Vaccine AE Propensity exhibited p <0.20 | | | | |

**Table S1a** shows the correlation of reported Vaccine AE Propensity to individual exposures. **Table S1b** shows correlations between Gulf War specific exposures and reported Vaccine AE Propensity, among cases only. The correlation in all participants is influenced by the fact that case status is linked to exposures, and also to reported Vaccine AE Propensity. The relationships within cases are more relevant. If the exposures correlation with reported Vaccine AE Propensity exhibited a p-value ≤ 0.2, the variable was retested, and added to the (later) main model. In each case, the variables retained in the main model maintained a relationship to reported Vaccine AE Propensity. Values in the far-right column display the p-values for the variable defining that row after addition to the main model, if applicable. Significance of most variables did not survive addition to the model (while a role for main model variables was sustained).

A 2004 paper focused on anthrax vaccination in the 1998 onward time frame (so, well after the Gulf War) reported low rates of serious adverse events (SAEs) and “other medically important adverse events (OMIAEs).” This is not inconsistent with reports in our sample of high anthrax vaccine adverse effects (AEs), for several reasons*:
(1) The vaccine lot testing (and site inspection) process was different later. (2) Their focus is on SAEs and OMIAEs, whereas we consider all reported AEs. (3) Even there, the low rates inferred require considerable interpretation, as ≥ 82% of AEs reported to the FDA’s vaccine adverse event reporting system (VAERS) were rejected as possible anthrax vaccine AEs, despite the submitter interpreting the AE as at least possibly related, and despite absence of a body of evidence to permit confident rejection of reported effects as possibly related to the anthrax vaccine. (4) *Overall* anthrax vaccine AEs have *not* been reported to be low. Studies, after the Gulf War, with active prospective follow up cite high rates of anthrax vaccine AEs (~41% to ~92%) ^1-5^. Our participants’ reported rate was 46% and this is in Veterans with Gulf War illness (GWI), a condition tied statistically to vaccine and specifically anthrax vaccine AEs, in whom rates of anthrax vaccine AEs may be expected to be higher ^6-8^. This does not support overreporting of vaccine AEs, or anthrax vaccine AEs, in our sample. (5) A published analysis of VAERS data reported that, by that index as well, the anthrax vaccine was among the most reactogenic of all vaccines ^9^, consistent with higher reports of AEs for anthrax than other vaccines in our sample. (6) Finally, importantly, the premise of the present analysis is that exposures may alter vaccine AE rates. (Clearly, something did.) Therefore, low rates from a sample that did not experience the abundance of Gulf-related exposures would not, in any case, have been inconsistent with materially higher rates in a sample that did experience such exposures.

Therefore, our participants’ apparent high rates of reported anthrax AEs are in no way inconsistent with the available literature.

*We thank a reviewer of this paper for suggesting inclusion of such information in the Supplement.

1. Hoffman K, Costello C, Menich M, Grabenstein JD, Engler RJ. Using a structured medical note for determining the safety profile of anthrax vaccine for US soldiers in Korea. Vaccine 2003;21:4399-409.

2. Pittman PR, Gibbs PH, Cannon TL, Friedlander AM. Anthrax vaccine: Short-term safety experience in humans. Vaccine 2002;20:972-8.

3. Pittman PR, Kim-Ahn G, Pifat DY, et al. Anthrax vaccine: Immunogenicity and safety of a dose-reduction, route-change comparison study in humans. Vaccine 2002;20:1412-20.

4. Wasserman GM, Grabenstein JD, Pittman PR, et al. Analysis of adverse events after anthrax immunization in US Army medical personnel. J Occup Environ Med 2003;45:222-33.

5. Gunzenhauser JD, Cook JE, Parker ME. Acute side effects of anthrax vaccine in ROTC cadets participating in advanced camp, Fort Lewis, 2000. MSMR 2001;7:9-11.

6. Unwin C, Blatchley N, Coker W, et al. Health of UK servicemen who served in Persian Gulf War. Lancet 1999;353:169-78.

7. Hotopf M, Hull L, Ismail K, Unwin C, Wessely S, David A. Vaccinations as a risk factor for ill health in Gulf War veterans: Results of the King’s study [abstract]. Proceedings of the Conference on Federally Sponsored Gulf War Veterans’ Illnesses Research; 1999 Jun 1999; Pentagon City. p. 103.

8. Schumm WR, Reppert EJ, Jurich AP, et al. Self-reported changes in subjective health and anthrax vaccination as reported by over 900 Persian Gulf War era veterans. Psychol Rep 2002;90:639-53.

9. Geier MR, Geier DA. Gastrointestinal adverse reactions following anthrax vaccination: an analysis of the Vaccine Adverse Events Reporting System (VAERS) database. Hepatogastroenterology 2004;51:762-7.
